# Author Correction: Dynamic patterns of YAP1 expression and cellular localization in the developing and injured utricle

**DOI:** 10.1038/s41598-021-03117-x

**Published:** 2021-12-08

**Authors:** Vikrant Borse, Matthew Barton, Harry Arndt, Tejbeer Kaur, Mark E. Warchol

**Affiliations:** 1 Department of Otolaryngology, School of Medicine, Washington University in Saint Louis, 660 South Euclid Ave, Box 8115, St Louis, MO 63110 USA; 2 Department of Biomedical Sciences, Creighton University School of Medicine, Nebraska, USA

Correction to: *Scientific Reports* 10.1038/s41598-020-77775-8, published online 25 January 2021

The original version of this Article contained errors in Figure 3E, where the image used for “3 mM Neomycin” was inadvertently swapped with the image used for the “Control.”

The original Figure 3 and accompanying legend appear below.

**Figure 3:**
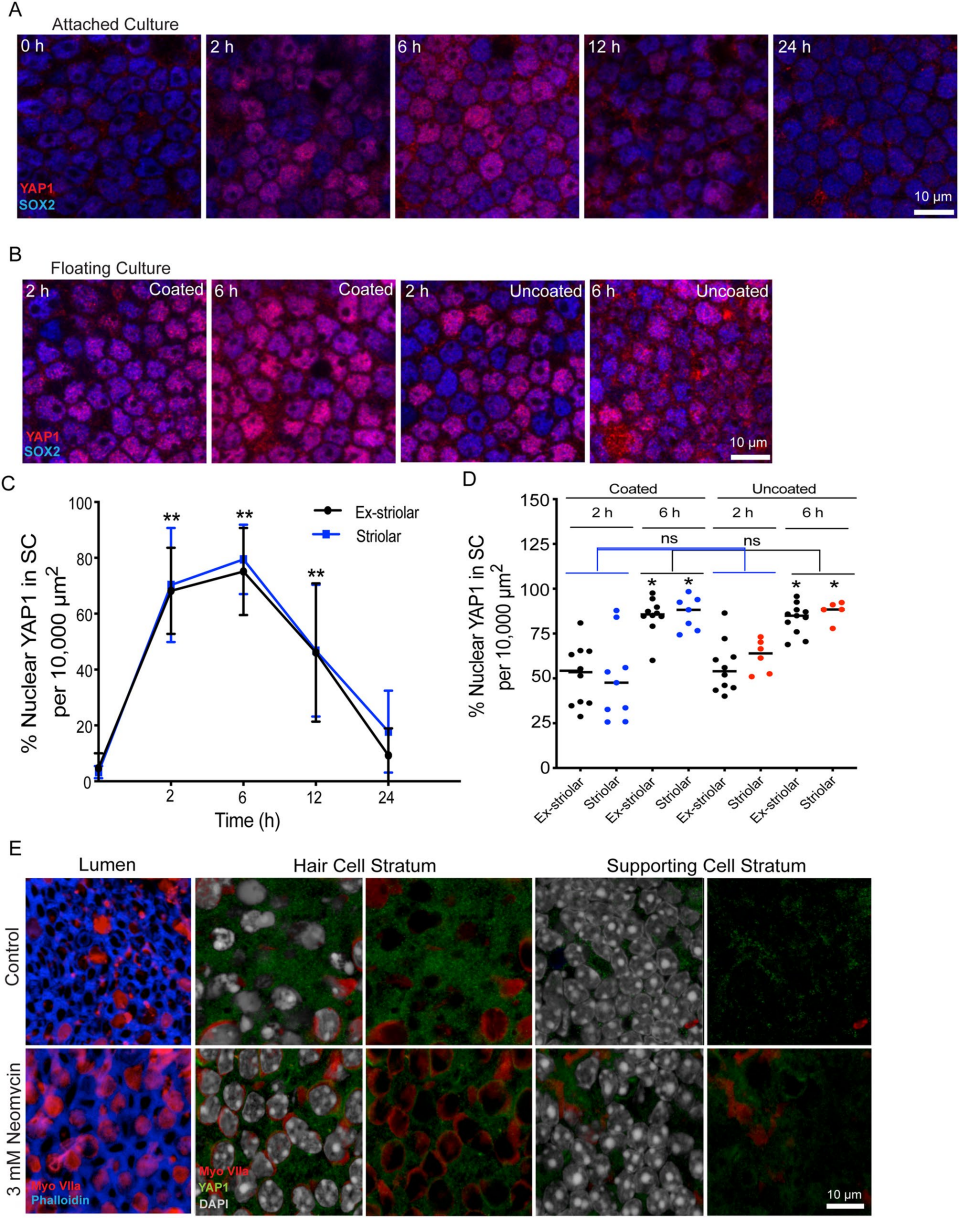
Transient nuclear translocation of YAP1 in organotypic culture of the mouse utricle. (**A**) Utricles were explanted from CD1 mice at P15, and attached to Matrigel-coated dishes. Specimens were fixed and examined after 0–24 h in culture. Confocal images show supporting cells of cultured utricles immunolabeled for YAP1 (red) and SOX2 (blue). (**B**) Utricles were explanted at P15 and cultured in a Matrigel-coated or uncoated dishes as free-floating samples. Cultures were fixed and examined after 2 and 6 h in vitro. Confocal images show supporting cells of cultured utricles immunolabeled for YAP1 (red) and SOX2 (blue). (**C**) Quantitative data on the percentage of supporting cells with nuclear YAP1 immunoreactivity. All data were obtained from 10,000 μm^2^ regions within the extrastriolar and striolar regions of each utricle. There was a significant increase in nuclear YAP1 immunolabeling at 2 h, 6 h and 12 h in vitro, relative to specimens fixed immediately after explantation (0 h) (p < 0.0001, 12 h, Ex-striolar p = 0.0027, Striolar p = 0.0198). (**D**) Quantitative data on the percentage of supporting cells with YAP1-labeled nuclei. All data were obtained from 10,000 μm^2^ regions within the extrastriolar and striolar regions of each utricle. There was no significant increase in the percentage of cells with nuclear YAP1 at 2 hr and 6 hr time points between the coated and uncoated cultures. However, there was a significant increase in nuclear YAP1 immunolabeling in utricles cultured in coated and uncoated dishes at the 6 hr time point, vs. those cultured for 2 hr (p < 0.0001). (**E**) Effects of culture in neomycin on YAP1 nuclear immunoreactivity in supporting cells. Images at far left show the lumen of the sensory epithelium in control and neomycin-treated utricles. Culture for 24 h in 3 mM neomycin led to reduced numbers of hair cells (red). Remaining images show z-sections through the sensory epithelia of control and neomycin-treated utricles, at the level of hair cell nuclei (middle images) and supporting cell nuclei (images at right). Each z-section is shown with and without DAPI-labeled nuclei (grey). Regardless of treatment condition, immunoreactivity for YAP1 (green) was primarily confined to the cytoplasm of supporting cells. Data expressed as mean ± SD. Statistical test was one-way ANOVA followed by Bonferroni’s post hoc test (*p value < 0.05) (**p-value < 0.05 for Ex-striolar and Striolar both region). N = 3–6 utricles.

The original Article has been corrected.

